# Assessing Consumer Behavior in the Wine Industry and Its Consequences for Wineries: A Case Study of a Spanish Company

**DOI:** 10.3389/fpsyg.2019.02491

**Published:** 2019-11-06

**Authors:** Rosa M. Muñoz, M. Valle Fernández, Maria Yolanda Salinero

**Affiliations:** ^1^Business Management Department, Group for Research in Organizational Knowledge, Innovation and Strategy, University of Castilla–La Mancha, Ciudad Real, Spain; ^2^Cofinanciación de la Unión Europea a Través del Fondo Europeo de Desarrollo Regional, Ciudad Real, Spain

**Keywords:** wine industry, consumer behavior, technology, digital transformation, case study

## Abstract

The widening gap between production and domestic consumption has led Spain to become the country with the largest volume of wine exports, of which it has a global market share of 20.5%. Wineries in Spain have, in recent years, undergone important transformation and modernization processes, while their customers have simultaneously acquired more power than ever before owing to both the availability of information and the technological means required to access that information. The objectives of this paper are the following. We first wish to analyze current wine consumer behavior in an attempt to discover indicators associated with exploratory behavior, i.e., the willingness to try new and innovative products for the first time. We also attempt to understand the extent of wine consumers’ demands in this new digital and technological world. The second goal is to analyze the changes that have taken place in the wine industry, which have come about as a result of the evolution of consumers’ expectations and demands. The customer accordingly now plays a new role in this new quick change scenario, which involves aspects such as the intensive use of technology, advances in communication, digital transformation, etc. We consider that this could affect some aspects of business and have, therefore, chosen to analyze one of the most advanced Spanish wineries in order to examine its entrepreneurial orientation, strategy and level of technological and digital transformation when linked to adaptation to this new consumer behavior. The results and conclusions obtained will allow us to apply our findings in future research, during which we intend to expand our studies to wineries from other regions and countries.

## Introduction

Wine is a product with an immediate effect on the consumer and a high level of differentiation, so customer orientation is a fundamental determinant of the competitive advantage of a company. Moreover, improving performance in markets for differentiated customer goods has been recognized as a major challenge for the European Union countries (Zaharieva et al., 2004).

The wine sector has, in recent years, undergone an evolution marked by an increase in market competition. In 2017, global wine production fell to 250 million hectoliters (mhl), a decline of 23.6 mhl when compared with 2016 production, while consumption increased a little to 243 mhl (OIV, 2018). A more detailed analysis shows that the difference between production and consumption is noticeable in traditional wine-producing countries (Lorenzo et al., 2018). For example, production in Italy, France and Spain in 2017 was 42.5, 36.7, and 32.1 mhl, respectively, while consumption was 22.6, 27.0, and 10.3 mhl. This important difference between production and domestic consumption has changed the way of competing in these countries, which have started to export in order to sell their products on international markets (Lorenzo et al., 2018). This widening gap between production and domestic consumption has made Spain the country with the largest volume of wine exports (22.1 mhl), with a global market share of 20.5% (OIV, 2018). Wineries in Spain have developed important processes of modernization and transformation during the last years. It is estimated that, since 2000, more than 130,000 hectares have been restructured and converted, with investments of more than 800 million euros (Lorenzo et al., 2018). Customers have simultaneously acquired more power than ever before owing to the availability of both information and the technological means required to access that information. It is, therefore, imperative to understand why and how consumers interact in this new environment (Szolnoki et al., 2014).

The objectives of this paper are the following. We first wish to analyze current wine consumer behavior in an attempt to discover indicators associated with exploratory behavior, i.e., the willingness to try new and innovative products for the first time. We also attempt to understand the extent of wine consumers’ demands in this new digital and technological world. The second goal is to analyze the changes that have taken place in the wine industry, which have been fostered by the evolution of consumers’ expectations and demands. The customer accordingly now plays a new role in this new quick change scenario, which involves aspects such as the intensive use of technology, advances in communication, digital transformation, etc. We consider that some aspects of business could be affected and have, therefore, chosen to analyze one of the most advanced Spanish wineries in order to examine its entrepreneurial orientation, strategy and level of technological and digital transformation when linked to adaptation to this new consumer behavior.

This paper is organized as follows. The following section draws from literature in the field, after which the methodology is explained. The “Case Study” section provides a description of the history of the firm and its main characteristics, along with an explanation of the strategy adopted, and the company’s technology and digital strengths, paying particular attention to its effect on the consumer-company relationship. Finally, the last section presents the main conclusions and implications extracted from the case study.

## Consumer Behavior in the Wine Industry and Its Consequences for Wineries

In the following sections, we present the main characteristics of wine consumer behavior and the company’s features that we consider most closely linked to them.

### Exploratory Consumer Behavior and Wine Consumption

The special characteristics of the wine industry create an ideal context in which to analyze exploratory consumer behavior. This concept emerged over six decades ago from the theory of optimum stimulation level. According to this theory, consumers seek to get an optimal level of stimulation from the environment, so that it is not too low that leads to boredom, nor so high that it becomes upsetting (Orth and Bourrain, 2005). Exploratory behavior occurs when consumers seek more stimulation by trying different or unusual products and find this exploration process enjoyable and rewarding. This could be denominated as innovativeness, i.e., when consumers expose to the risk of using a previously unknown product, variety seeking or switching behavior, i.e., when they avoid the boredom that might occur as a result of repeatedly consuming the same product; and curiosity-motivated, when motivates consumer’s information search about new or unfamiliar products. We focus on the first form of exploratory behavior, which is to say, the search for new wines for personal consumption (Schaefer et al., 2018). One of the studies related to this issue found that consumers who showed an exploratory behavior, tended to buy greater quantities of wine, drank wine more frequently, were more highly interested in the wine category, and had greater knowledge of this sector (d’Hauteville and Goldsmith, 1998).

### Entrepreneurial Orientation

Entrepreneurial orientation (EO) is a company decision-making proclivity that favors entrepreneurial initiatives and there is an agreement that it represents a continuous variable in which all enterprises can be positioned (Lumpkin and Dess, 2001). Some authors explore how this concept has been studied and assessed in prior research (Covin and Wales, 2012). They state that authors are free to choose whichever measurement approach best serves their research objectives. In this respect, some researchers have considered EO as a variable composed of three sub-dimensions: innovativeness, risk taking and proactiveness (Miller, 1983; Covin and Slevin, 1989).

### Company Strategy

One of the models that has been used in an attempt to capture the typology of the competitive strategy is that developed by Robinson and Pearce (1988), and it has been used in several studies (Ibrahim et al., 2001; Spanos and Lioukas, 2001; Ruiz Ortega, 2010). This model was developed on the basis of previous studies by Dess and Davis (1984) and looks for expanding the generic strategies of Michael Porter, thus favoring their characterization in empirical business studies. This business strategy proposal consists of 22 indicators assessed using a five-point Likert scale, on which firms evaluate themselves with respect to different company development efforts, from 1 “not considered” to 5 “major, constant emphasis.”

### Technology

In the case of the technology employed in this industry, wineries have started to develop innovative *traceability* tools to protect their image against the negative consequences of counterfeit wines and to strengthen their brand equity (Wang et al., 2017). There are various kinds of voluntary traceability in the wine sector, such as the ISO 22005 and the private standards that are adopted by operators like wineries or retailers (Stranieri et al., 2018). With regard to the motivations leading wineries′ decisions to develop voluntary quality standards, most of the studies identify firm internal efficiency, and external business factors, such as customer requirements (Corsinovi and Gaeta, 2017). Heightened customer demand for higher quality and standardized products is a primary driver of this (Matthew Rendleman et al., 2016). The implementation of a quality control program results in an increased reputation and production value. The objective of supply chain traceability is to identify the economic agents of the supply chain. This system is mandatory in Europe. However, it does not allow the traced information to be associated with a specific product, and does not provide a reconstruction of the product history. Supply chain and traceability of products entails more complex systems. They are characterized by the management of products and raw materials in separate lots and by procedures that assign specific information to each of said lots (Stranieri et al., 2018).

With regard to technology, we also wish to examine the adoption of intelligent automation (IA), which makes the consumer-companies relationships easier. It includes: robotic process automation (RPA), artificial intelligence (AI), machine learning (ML), cognitive computing (CC) and smart analytics (KPMG, 2018). IA can give companies a competitive advantage in all aspects of business.

### Digital Transformation

Digital transformation is another relevant issue. The adoption of the Internet has become an important aspect of the renewal process required by the wine industry. It has moved rapidly into a new step of consumer interaction and influence via social networks, blogs, podcasts, and online virtual communities. This fact has implications for many sectors, but the wine industry is one that is particularly suited to this new tendency (Thach, 2009).

Social media have grown dramatically over the past decade, signifying that it is important for firms to learn how to interact with consumers via these new channels. This phenomenon has significantly altered the way in which customers communicate and interact with each other and with companies (Siamagka et al., 2015). This is particularly problematic in the consumer product sector, because consumers communicate about brands online, regardless of whether those brands respond or not. A lack of response in public social media can harm companies (Szolnoki et al., 2018). Social media have consequently led to a shift in power to consumers, as they move from passive receivers of marketing strategies to active participants in the brand message (Mangold and Faulds, 2009). Social media usage has been found to have a positive effect in brand performance and customer loyalty (Rapp et al., 2013).

The global wine industry is one of the industries whose brand is the subject of global online conversations (Mueller et al., 2010). Earlier literature has suggested that wineries that have a presence in social media will achieve low-cost benefits such as increased sales through word of mouth, loyalty and ongoing relationships with customers (Humphrey et al., 2017). The socialization aspect of social media allows consumers to exchange information and encourage others to try different wines, signifying that it is a key channel as regards influencing and affecting the purchase of wine (Wilson and Quinton, 2012).

Wineries, along with most other major industries, began to incorporate the Internet into their business strategies in the 1990s. The questions that should be addressed are, however, how effective is their communication with consumers online? And are wineries adopting the new Wine 2.0 philosophy of reaching out to consumers and actively engaging with them? (Thach, 2009).

The term Wine 1.0 refers to wine being featured on the Internet in a passive fashion, such as on a basic non-interactive website that includes general information on the company, its products, and contact data. The term Wine 2.0 refers to using the Internet to engage with wine consumers on their terms, in a time and manner of their choosing, and tools usually include social networking sites, blogs, message boards, and interactive components (Olsen and Hermsmeyer, 2008). Wine 3.0 is a phenomenon that includes virtual reality wine tasting, avatars, or wine reviews that are accessible via cell phones and bar codes on wine bottles (Thach, 2009). Certain product categories, such as wine, require knowledge and information in order to decide to buy them, and recommendations work well. Therefore, components such as blogs and social networking sites may influence wine purchasers. Nowak and Newton also found that “website quality, defined as a professional-looking website with user-friendly menus and prompts, was a significant predictor of increased trust in the winery and perceptions of the quality of the wine” (Nowak and Newton, 2008).

## Methodology

The wine sector has been studied from different points of view in literature. Studies regarding the United States wine industry are focused on the relationship between differentiation strategies and performance (Newton et al., 2015). In terms of strategy, there is research concerning the marketing strategy and performance in the French wine sector (Hammervoll et al., 2014), while in Spain, some authors have analyzed the strategies, environmental variables, and economic performance (Simon-Elorz et al., 2015). Other works have focused on the internationalization capacity in the Italian wine sector (Galati et al., 2016), or on cluster resources and performance in the Brazilian wine industry (Fensterseifer and Rastoin, 2013). This paper adopts an exploratory perspective and employs a qualitative approach through a case study. It was used to gain deeper insights into a complex issue within its real-life context. The objective of case-based research is to produce a refined theory based on an in-depth understanding of a particular context (Gerring, 2007). The single case setting limits the applicability of the research to other institutions. However, the framework and model that are developed, along with the overall approach, are valuable contributions to an important and emerging research area. The “how” and “what” questions are asked about a contemporary set of events over which the investigator has little or no control (Whitehead and Yin, 2003). This study investigates “how” wine consumers behave nowadays and “what” changes are observed in wineries as a result. As the paper seeks to address research questions, this suggests the adoption of an exploratory approach, in the sense that the main objective is to refine a research idea in order to facilitate further research. Study conducted within the qualitative paradigm is characterized by its commitment to collecting data from the context in which social phenomena naturally occur and to generating an understanding that is grounded on the perspectives of research participants (Marshall and Rossman, 1995).

The qualitative approach and exploratory nature of the research question influenced the data-collection method. It was consequently collected using desk and field research. Desk research, which is based on studying internal company documents, served to provide a detailed understanding of the company’s situation, while the field research included a tour of the company visiting all departments, a questionnaire and an in-depth interview with the company’s Managing Director. The interview took place in April–May 2019. It lasted 90 min and was tape-recorded, immediately transcribed and then sent back to the interviewee for review to ensure “truth value.”

We have included the following dimensions in our research: exploratory consumer behavior, EO, company strategy and technological and digital transformation. Robinson and Pearce (1988), Covin and Slevin (1989), and Schaefer et al. (2018) scales were chosen in order to analyze the exploratory consumer behavior, the EO and the company strategy in our case study (see Appendix, the Managing Director’s answers indicated with x). Besides the information in the Appendix, the interview included questions related to the company adoption of intelligent automation and its Wine 2.0 components. In the following section, we describe the winery, its activity and its most recent innovations. We then explain the principal ideas extracted from the data analyzed, the interview and the questionnaire.

## Case Study

Vinícola de Castilla S.A. is a Spanish winery located in the city of Ciudad Real whose activity started in 1976. Its products are sold in 42 countries around the world. Some data related to the company are shown in Table 1.

**TABLE 1:** Data regarding Vinícola de Castilla.

**Variable**	**2015**	**2016**	**2017**
Turnover (mill. €)	4.6	5.2	4.2
Number of employees	30	28	27
Solvency ratio %	23	17	17
Liquidity ratio %	17	12	10

*Vinícola de Castilla’s Annual Reports.*

These data show that the firm has a healthy financial and economic situation with good levels of growth and profit. The decrease observed in the ratios is a consequence of the strong investments in technology and digitalization that the company is carrying out in recent years.

With a speed of 15,000 bottles/hour and in a continuous updating and optimization process, the bottling line has the most advanced machinery with which to attain the safest presentation that is of the highest quality. The boxing and palletizing systems, which are completely customizable, provide many possibilities as regards the final packaging process of products. All the area systems are controlled by Programmable Logic Controllers (PLCs) and industrial computers. The facilities are airy, well-equipped and illuminated for industry-leading machines. A team of enologists monitors the entire elaboration process, from harvesting to bottling, thus ensuring that the product has the best characteristics. Traditional and modern methods coexist, and a full range of tools is available for monitoring, traceability and strict quality control. Vinícola de Castilla has its own vineyards, which are divided mainly into two types: bush vines and espalier. The quality and high food security procedures are certified by very prestigious authorities under the most demanding standards.

### Exploratory Consumer Behavior

As has occurred in other recent studies (Thach et al., 2015), exploratory consumer behavior was measured using the variety seeking scale that (Schaefer et al., 2018) adapted for wine (see Appendix). We have adapted the questions in order to enable them to be answered by the manager of the company. That is to say, we wished to discover his perceptions of Vinícola de Castilla consumers’ exploratory behavior. All seven items used were measured on a five-point Likert type scale. The individual items on the scale best capture aspects of exploratory behavior, including curiosity and innovativeness.

The Vinícola de Castilla consumers have low levels of exploratory behavior: they do not try unusual or exotic wines; they prefer the wines they are used to, and they do not like to try wines from different countries.

### Entrepreneurial Orientation

According to the Covin and Slevin scale (Covin and Slevin, 1989) it is possible to consider that the firm has an EO, i.e., it has high levels of innovativeness, proactiveness, and risk taking. We can, in general terms, summarize that the company has, over the last few years, marketed many new lines of products and has placed a particularly strong emphasis on the marketing of tried-and-true products. When dealing with its competitors, it typically starts actions to which competitors then respond, and it is very often the first firm to introduce new products, processes and operating technologies, adopting a competitive posture. The company’s top managers claim that, owing to the nature of the environment, bold, aggressive acts are necessary to achieve the firm’s objectives.

### Company Strategy, Technology, and Digital Transformation

When considering the items included in the business’s company scale (see Appendix), the company appears to have a remarkable orientation toward the market as a source of its competitive advantage, and this is integrated into a clear strategy of *differentiation*. The winery focuses on the marketing function in an attempt to achieve an identifiable brand and a reputation in the market. It relies heavily on *innovation in methods and marketing techniques*. In this respect, the company has developed a marketing plan with which to enhance its online presence. It participates in trade journeys and commercial fairs to a significant extent, and has received more than 700 prestigious national and international awards and recognitions (one of its best products has recently been recognized with the prestigious Golden Bacchus 2019), and this is the best form of publicity for the company.

Differentiation through the recognition of its brand image is the result of its tradition and image of *quality*, which are reflected in its commitment to the continuous improvement of its processes and products and the great efforts made to obtain the best raw materials. Its Methods and Quality Control Department applies a policy of maximum quality.

The company has the best and most innovative technical-enological endowment, thanks to its heavy investment in technology and innovation that allows it to compete, simultaneously, via quality and via costs.

*Customers service* is another of the significant aspects of the winery’s business strategy, since it attempts to provide its customers with after-sales service and support, in addition to offering them high food safety, which is certified by entities of recognized prestige under the most demanding standards. The company has a complete *traceability system*: it has had ISO 9001 records for more than twenty years. Its scope of certification is: the design, elaboration, aging, bottling and commercialization of wines, and it also has the BRC Global Standard for Food Safety maximum level certifications and the International Featured Standard (IFS) Food for the safety and quality of food products and production processes. With regard to the consumer, the BRC Global Standard establishes customer focus and communication, that is to say, the need to ensure that customer requirements are met and that these requirements are communicated to relevant suppliers.

Flexibility and adaptation are also remarkable company characteristics. The winery has the capability to create *new specialized products*, i.e., the company has been the first winery in Castilla-La Mancha to produce certain special products, such as distilled beverages made from wine (Olimpo Holanda) or natural drinks derived from wine (Dlavid).

In the case of intelligent automation, Vinícola de Castilla is starting to develop certain processes, such as RPA and artificial intelligence (specifically, predictive and prescriptive analysis), but they are still in the first stage.

Upon examining its digital transformation we discovered that the company utilizes the majority of the components of Wine 2.0: a website with an e-commerce engine that is expanded to include interactive components, a wine blog, social networking sites (Twitter, Pinterest, and Facebook linked to the website), and winevlogs, that is, a video placed on its website in order to entertain customers and educate them about the company and its products.

## Conclusion

Marketing literature suggests that a company’s long-term success and survival is dependent on a firm’s ability to become consumer rather than product-oriented (Greenley et al., 2003). This paper analyses a winery’s efforts to achieve consumer orientation by means of strategy and technology. The main ideas and suggestions extracted from the analysis are shown as follows.

With regard to the first objective, that is to say, the analysis of current wine consumer behavior, we can conclude that the Vinícola de Castilla *consumers* have low levels of *exploratory behavior*, which is possibly related to the fact that 50% of the company sales are in the national market and 25% are in the European Union. That is to say, only 25% are in the rest of the world. The consumers who are most likely to engage in exploratory behavior value creativity, risk taking and also have a more global outlook, as they are more likely to purchase wine from other countries (Schaefer et al., 2018). We suggest that the company focuses on consumers with an exploratory behavior in line with its advances in technology and digitalization which entails a greater internationalization. The company should keep in mind the two main trends that will influence the wine sector in the future and characterize consumers’ choices: most of them will still look for the best value for money and the best deals, but there will be a growing group of sophisticated and expert wine customers who will want experience more and look for high quality products and brands with high-end attributes (KPMG, 2014). Vinícola de Castilla should consider ways in which to expand its brand portfolio to reach more sophisticated wine drinkers both in Spain and worldwide.

In the case of the second objective, that is to say, the analysis of the business changes linked to the adaptation to consumer behavior, we present some suggestions. With regard to *new technologies*, Vinícola de Castilla is not exploiting all its intelligent automation potential. It is now starting to employ RPA and artificial intelligence, but it should also consider the future implantation of certain technologies such as computer vision or machine learning. These technologies enable systems to automatically learn and improve from experience without being explicitly programed. Computer vision is concerned with the theory behind artificial intelligence that extracts information from images. Machine learning focuses on the development of computer programs that can access data and use it to learn for themselves. All these technologies are appropriate for the wine sector and could improve both the processes in wineries and their relationships with consumers.

The Vinícola de Castilla *digital transformation* is in line with consumers’ current demands. In this respect, the company has developed most of the components of Wine 2.0., but it should also start paying attention to the most advanced components of Wine 3.0, such as wine reviews that are accessible via cell phones and bar codes on wine bottles, virtual reality wine tasting and avatars. Some possibilities include, for example, creating an avatar that looks like the winemaker, and incorporating professional wine critic ratings with customer ratings of the wine brand. This could give the company a competitive advantage as a first mover.

Vinícola de Castilla has several strengths that have placed it in a good competitive position in the last few years:

• an entrepreneurial orientation characterized by high levels of innovativeness, proactiveness, and risk taking;
• a differentiation strategy;
• consumer service orientation;
• well-established quality and traceability systems, along with the latest technological trends as regards processing and communication.

Besides these strengths, the company should, considering the future scenario, concentrate on some important issues such as exploratory consumer behavior, new technologies related to intelligent automation, and Wine 3.0.

This paper has several limitations that show possible lines for further research. It has developed an in-depth case study to a company. Case studies are a detailed contextual analysis of a limited number of events or situations and their relationships. The findings should, therefore, be treated with caution and should be checked and validated in other companies and other regions and countries. Various future research opportunities may, therefore, be possible. Despite these limitations, the results are relevant and provide some key implications for managers and researchers and new issues for future studies. In relation to what is learned from this study, we can ask ourselves, to what extent are these results applicable to other organizations in the wine industry? We can affirm that the analyzed company is one of the most advanced in its sector and most wineries are also smaller. They have to catch up on issues such as digitalization and technology if they really want to respond to the consumer behavior as Vinícola de Castilla is doing.

## Data Availability Statement

All datasets generated for this study are included in the article/Supplementary Material.

## Author Contributions

RM has made the theoretical review and empirical study. MF has developed the interviews with the company’s CEO and made the empirical study. MS has made the empirical study.

## Conflict of Interest

The authors declare that the research was conducted in the absence of any commercial or financial relationships that could be construed as a potential conflict of interest.
