# Supplementary material for: Assessing Consumer Behavior in the Wine Industry and Its Consequences for Wineries: A Case Study of a Spanish Company
Source: Front Psychol. 2019 Nov 6;10:2491. doi: 10.3389/fpsyg.2019.02491 (PMC6851156; doi:10.3389/fpsyg.2019.02491)
Supplement: Supplementary file 1 [file Data_Sheet_1.docx]

**APPENDIX**

**INTERVIEWS ITEMS**

**Measures of exploratory behavior as regards wine**

|  | Completely desagree | Completely  agree |
| --- | --- | --- |
| Vinícola de Castilla consumers like to try the most unusual wines, even if they are not sure they would like them | ①             ② x            ③         ④             ⑤ | |
| Vinícola de Castilla consumers think it is fun to try out the wines they are not familiar with | ①             ②   x          ③         ④             ⑤ | |
| Vinícola de Castilla consumers like to drink exotic wines | ① x            ②             ③         ④             ⑤ | |
| Vinícola de Castilla consumers like to try wines from different countries | ➀ x            ➁             ③         ④             ⑤ | |
| Vinícola de Castilla consumers are constantly sampling new and different wines | ➀ x            ➁             ③         ④             ⑤ | |
| Vinícola de Castilla consumers prefer to drink only the wines they are used to (Reverse scored) | ➀             ➁             ③         ④ x            ⑤ | |
| Vinícola de Castilla consumers are afraid to try wines they have never had before (Reverse scored) | ➀             ➁             ③ x        ④             ⑤ | |

Source: adapted from (Schaefer, Olsen, and Thach 2018)

**Entrepreneurial orientation**

| INNOVATIVENESS ITEMS |  |  |  |  |  |  |  |  |
| --- | --- | --- | --- | --- | --- | --- | --- | --- |
| In general, the top managers of my firm favor… |  |  |  |  |  |  |  |  |
| A strong emphasis on the marketing of tried-and-true products of services | 1 | 2 | 3X | 4 | 5 | 6 | 7 | A strong emphasis on R&D technological leadership, and innovations |
| How many new lines of products or services has your firm marketed in the past five years (or since its establishment)? |  |  |  |  |  |  |  |  |
| No new lines of products or services | 1 | 2 | 3 | 4 | 5 | 6X | 7 | Very many new lines of products or services |
| Changes in product or service lines have been mostly of a minor nature | 1 | 2 | 3 | 4X | 5 | 6 | 7 | Changes in product or service lines have usually been quite dramatic |
|  |  |  |  |  |  |  |  |  |
| PROACTIVENESS ITEMS |  |  |  |  |  |  |  |  |
| When dealing with its competitors, my firm… |  |  |  |  |  |  |  |  |
| Typically responds to actions that competitors initiate | 1 | 2 | 3 | 4 | 5 | 6X | 7 | Typically initiates actions to which competitors then respond |
| Is very seldom the first business to introduce new products/services, administrative techniques, operating technologies, etc | 1 | 2 | 3 | 4 | 5 | 6X | 7 | Is very often the first business to introduce: new products/services, administrative techniques, operating technologies, etc. |
| Typically seeks to avoid competitive clashes, preferring a “live-and-let-live” posture | 1 | 2 | 3 | 4 | 5 | 6X | 7 | Typically adopts a very competitive, “undo-the-competitors” posture |
|  |  |  |  |  |  |  |  |  |
| RISK-TAKING ITEMS |  |  |  |  |  |  |  |  |
| In general, the top managers of my firm have… |  |  |  |  |  |  |  |  |
| A strong proclivity for low-risk projects (with normal or and certain rates of return) | 1 | 2 | 3X | 4 | 5 | 6 | 7 | A strong proclivity for high-risk projects (with chances of very high returns) |
| In general, the top managers of my firm believe that… |  |  |  |  |  |  |  |  |
| Owing to the nature of the environment, it is best to explore it gradually via cautious, incremental behavior | 1 | 2 | 3 | 4 | 5X | 6 | 7 | Owing to the nature of the environment, bold, wide-ranging acts are necessary to achieve the firm´s objectives… |
| When confronted with decision-making with situations involving uncertainty, my firm… |  |  |  |  |  |  |  |  |
| Typically adopts a cautious, “wait-and-see” posture in order to minimize the probability of making costly decisions | 1 | 2 | 3 | 4X | 5 | 6 | 7 | Typically adopts a bold, aggressive posture in order to maximize the probability of exploiting potential opportunities |

Source: (Jeffrey G. Covin and Wales 2012)

**Company strategy**

|  | Not considered | Very limited emphasis | Some emphasis | Considerable emphasis | Major, constant emphasis |
| --- | --- | --- | --- | --- | --- |
| Product quality | 1 | 2 | 3 | 4 | 5x |
| Development of new products | 1 | 2 | 3 | 4x | 5 |
| Business productivity | 1 | 2 | 3 | 4x | 5 |
| Continuing, overrinding convern for lowest cost per unit | 1 | 2 | 3 | 4 | 5x |
| Extremely strict product quality control procedures | 1 | 2 | 3 | 4 | 5x |
| Price | 1 | 2 | 3x | 4 | 5 |
| Wide range of products | 1 | 2 | 3 | 4x | 5 |
| Builds brand identification by the customer | 1 | 2 | 3 | 4x | 5 |
| Strong influence over channels of distribution | 1 | 2 | 3x | 4 | 5 |
| Major effort to ensure availability of raw materials | 1 | 2 | 3 | 4 | 5x |
| Innovation in manufacturing process | 1 | 2 | 3 | 4x | 5 |
| Customer service capabilities | 1 | 2 | 3 | 4 | 5x |
| Specific efforts to ensure a pool of highly trained experienced personnel | 1 | 2 | 3 | 4 | 5x |
| Maintains low inventory levels | 1 | 2 | 3 | 4x | 5 |
| Develops and refines existing products | 1 | 2 | 3 | 4 | 5x |
| Innovation in marketing techniques and methods | 1 | 2 | 3 | 4x | 5 |
| Promotion advertising expenditures above the industrial average | 1 | 2 | 3x | 4 | 5 |
| Emphasis on the manufacture of specialized products | 1 | 2 | 3 | 4 | 5x |
| Only serves specific geographic markets | 1 | 2 | 3 | 4x | 5 |
| Specialization in products in higher priced market segments | 1 | 2 | 3x | 4 | 5 |
| Major effort to improve the quality of advertising | 1 | 2 | 3x | 4 | 5 |
| Concerted effort to build reputation within industry | 1 | 2 | 3 | 4 | 5x |

Source: (Robinson and Pearce 1988).
